# Supplementary material for: Delays in obtaining hospital care and abortion-related complications within a context of illegality
Source: PLoS One. 2023 Jun 14;18(6):e0286982. doi: 10.1371/journal.pone.0286982 (PMC10266605; doi:10.1371/journal.pone.0286982)
Supplement: Attachment — Submitted filename: Comments.docx [file pone.0286982.s001.docx]

**Title: Delays in obtaining hospital care and abortion-related complications within a context of illegality**

This is a further analysis of a study conducted in 2010, it contributes to the understanding of the delay in performing uterine evacuation and occurrence of abortion-related complications.

The author has provided reasonable explanation regarding the restrictions in availability of data in the manuscript (review question (3)).

Comments:

1. Page 5 - Introduction

I suggest including information regarding the legal status of abortion in the areas where the study was conducted as it is not clear. There is a statement on regions with restrictive legislation in line 74, however, it is not clear whether this include the regions of study. The meaning of restrictive legislation for abortion needs to be explained.

2. Page 9, line 178:

The statement ‘to calculate the number of losses’ is not clear; does ‘number of losses’ means ‘missing data’? This need clarification.

3. Tables 1 and 3:

To state the exact number of indigenous women and those of Asian descent which were excluded.
