# Supplementary material for: Delays in obtaining hospital care and abortion-related complications within a context of illegality
Source: PLoS One. 2023 Jun 14;18(6):e0286982. doi: 10.1371/journal.pone.0286982 (PMC10266605; doi:10.1371/journal.pone.0286982)
Supplement: Attachment — Submitted filename: Response to reviewers.docx [file pone.0286982.s002.docx]

**Response to reviewers**

Surangi Nilanka Jayakody Mudiyanselage, MBBS, MD

Academic Editor - PLOS ONE

Ref.: Delays in obtaining hospital care and abortion-related complications within a context of illegality (PONE-D-22-23361)

Dear Dr. Mudiyanselage

Thank you for having considered our manuscript for publication and for the reviewers’ helpful suggestions, all of which have been taken into consideration in this amended version of the paper. Please find below our point-by-point answers to the reviewers’ comments.

We are very grateful for the opportunity to resubmit this manuscript and hope that it will now be deemed suitable for publication.

Please do not hesitate to contact me again should you require any further information.

Yours sincerely

Romina M Hamui

In response to the academic editor’s suggestions:

*1. Please ensure that your manuscript meets PLOS ONE style requirements, including*

*those for file naming. The PLOS ONE style templates can be found at*

*https://journals.plos.org/plosone/s/file?id=wjVg/PLOSOne_formatting_sample_main_b*

*ody.pdf and*

*https://journals.plos.org/plosone/s/file?id=ba62/PLOSOne_formatting_sample_title_aut*

*hors_affiliations.pdf.*

The text has been reviewed once again to ensure that we meet with your requirements and to the best of our knowledge we believe that we have complied with the PlosOne guidelines

*2. Thank you for stating in your Funding Statement:*

*Please provide an amended statement that declares *all* the funding or sources of support (whether external or internal to your organization) received during this study, as detailed online in our guide for authors at http://journals.plos.org/plosone/s/submit-now.*

*Please also include the statement “There was no additional external funding received for this study.” in your updated Funding Statement.*

*Please include your amended Funding Statement within your cover letter. We will change the online submission form on your behalf.*

An amended statement has now been included in the cover letter, as requested:

**Funding statement:**

*This study received financial support from the Brazilian Ministry of Health (DECIT) and the Ministry of Science and Technology - National Council for Scientific and Technological Development (CNPq), grants MCT/CNPq/MS-SCTIE-DECIT/CT - Health 22/2007 (#551249/2007-2) and MCT/CNPq/MS/ SCTIEDECIT 54/2008 (#402680/2008-1). In addition, EMLA received a CNPq research productivity grant (306295/2017-2). There was no additional external funding received for this study.*

**URLs to sponsors’ websites:**

<https://www.gov.br/saude/pt-br>

<https://www.gov.br/mcti/pt-br>

<https://www.gov.br/cnpq/pt-br>

*The funders had no role in the study design, data collection and analysis, decision to publish, or preparation of the manuscript.*

3. *Please review your reference list to ensure that it is complete and correct. If you have cited papers that have been retracted, please include the rationale for doing so in the manuscript text, or remove* these *references and replace them with relevant current references. Any changes to the reference list should be mentioned in the rebuttal letter that accompanies your revised manuscript. If you need to cite a retracted article, indicate the article’s retracted status in the References list and also include a citation and full reference for the retraction notice.*

The reference list has been checked as requested. Two of the references have been updated:

*6 - Shah I, Ahman E. Unsafe abortion in 2008: global and regional levels and trends. Reprod Health Matters. 2010;18:90-101. https://doi.org/10.1016/S0968-8080(10)36537-2.*

*27 - Goes EF, Menezes GMS, Almeida MC, Araújo TVB, Alves SV, Alves MTSSBE, et al. Racial vulnerability and individual barriers for Brazilian women seeking first care following abortion. Cad Saude Publica. 2020;36(Suppl 1):e00189618. https://doi.org/10.1590/0102-311X00189618.*

In response to Reviewer 1:

*1. Page 5 - Introduction*

*I suggest including information regarding the legal status of abortion in the areas where the study was conducted as it is not clear. There is a statement on regions with restrictive legislation in line 74, however, it is not clear whether this includes the regions of study. The meaning of restrictive legislation for abortion needs to be explained.*

We are grateful to the reviewer for this suggestion. Text has now been added to the Introduction, as follows (Page 5, lines 72-74):

*Abortion is illegal in Brazil except when performed to save the woman's life or in cases of rape. In 2012, the Brazilian Supreme Court authorized abortion in cases of fetuses with anencephaly.*

*2. Page 9, line 178:
The statement ‘to calculate the number of losses’ is not clear; does ‘number of losses’ means ‘missing data’? This need clarification.*

We are particularly grateful for this comment as it gave us the opportunity to correct and provide a clearer explanation regarding these losses. To identify those eligible for the survey, the women admitted daily to each of the hospitals involved in the study were checked and a list of those to be interviewed by the investigators was prepared. A total of 2,848 interviews were carried out, with 5.8% of losses and 2.7% of refusals. This information has now been added to the text. (Page 9, lines 184-185)

*3. Tables 1 and 3:
To state the exact number of indigenous women and those of Asian descent, which were excluded.*

Thank you for this suggestion. The information requested has now been added to page 12, lines 243-245 and as a footnote to both tables, as follows:

*Seventy-six indigenous women (2.8%) and 69 women of Asian descent (2.5%) were excluded due to their small numbers and due to the presence of differences that would make it difficult for them to be included in any other group.*

In response to Reviewer Nº2:

1) *The authors of this manuscript have described " Delays in obtaining hospital care and abortion-related complications within a context of illegality" technically sound manner. However, the original study was conducted in 2010 which is more than 12 years back. Publishing an article and applicability of the recommendations based on a study which was conducted more than 12 years back is arguable.*

Thank you for this comment. The following text has now been added to the Conclusion:

*Abortion continues to be illegal in the country, with the only change being the incorporation by the Supreme Court in 2012 of a third legal exception to the law – in cases of anencephaly. Therefore, no change has occurred in unsafe abortion care within the public health network.*

2) *Table 2 is on time interval between admission and uterine evacuation according to the occurrence of complications. If the data are being skewed, better explain with median and IQR rather than explaining all mean, SD and median. Also author can mention the number of cases outside from this table*

Thank you for pointing this out. The Shapiro-Wilk test was performed and the distribution of the data is indeed not normal. Therefore, results have now been reported as medians. Means and SD have been removed from the table, as suggested. (Page 16).

3) *Table 3 - If Asian and Indigenous women were excluded, under ethnicity author can mention the number as n = .......*

Thank you for this suggestion. This information has now been added to the Results section (page 12, lines 243-245) and as a footnote to Tables 1 and 3, as follows:

*Seventy-six indigenous women (2.8%) and 69 women of Asian descent (2.5%) were excluded due to their small numbers and due to the presence of differences that would make it difficult for them to be included in any other group.*

4) The authors have included the recommendations also under conclusion. Better if the limitations have been mentioned.

We chose to present the limitations and strengths of the study in the Discussion (lines 479-498), reserving the summary of the main findings and their implications for future research and the reorganization of abortion care for the Conclusions. We would prefer to retain the manuscript in this format; however, should the editor deem it necessary, we are willing to make the changes suggested by the reviewer.
